# IDO Activation Affects BDNF/TrkB Signaling Pathway, Oxidative Stress, and Mitochondrial Enzymatic Activities in Temporal Lobe Epilepsy

**DOI:** 10.3390/cimb47090764

**Published:** 2025-09-16

**Authors:** Jingwen Xu, Liping Wei, Junling Fu, Ziting Kong, Lun Cai

**Affiliations:** 1 Department of Neurology, The First Affiliated Hospital of Guangxi University of Chinese Medicine, Guangxi University of Chinese Medicine, Nanning 530023, China; xujingwen2023@stu.gxtcmu.edu.cn (J.X.); fujunling2024@stu.gxtcmu.edu.cn (J.F.); kongziting2024@stu.gxtcmu.edu.cn (Z.K.); 2 Guangxi Key Laboratory of Molecular Biology of Traditional Chinese Medicine and Preventive Medicine, Nanning 530023, China; 3 Department of Rehabilitation, The First Affiliated Hospital of Guangxi University of Chinese Medicine, Guangxi University of Chinese Medicine, Nanning 530023, China; 13978856423@163.com

**Keywords:** temporal lobe epilepsy, indoleamine 2,3-dioxygenase, BDNF/TrkB, oxidative stress, mitochondrial respiratory chain complex

## Abstract

Indoleamine 2,3-dioxygenase (IDO) activation by seizures elevates toxic tryptophan metabolites linked to seizure exacerbation. Brain-derived neurotrophic factor (BDNF)/tyrosine kinase B (TrkB) signaling, oxidative stress, and mitochondrial respiratory chain complex dysfunction contribute to temporal lobe epilepsy (TLE), but their regulatory links remain unclear. Male Kunming mice were grouped into Control, Control + 1-Methyl-DL-tryptophan (1-MT), TLE, and TLE + 1-MT. TLE was induced with 300 mg/kg pilocarpine. Two weeks after modeling, 1-MT (50 mg/kg) was administered twice daily for two weeks in 1-MT groups. Assessments included video monitoring to record seizure frequency and duration; Nissl and Fluoro-Jade B (FJB) staining to evaluate neuronal damage; real-time quantitative PCR (qRT-PCR) and Western blot to detect IDO, BDNF, and TrkB expression; assays for the following oxidative stress markers: malondialdehyde (MDA), glutathione (GSH), superoxide dismutase (SOD), catalase (CAT); and detection of mitochondrial complex I/IV activities. Results showed TLE mice had significantly increased IDO expression, BDNF/TrkB over-activation, elevated oxidative stress, impaired mitochondrial complex I/IV activities, severe neuronal damage, and increased seizure frequency/duration. 1-MT intervention reversed all these pathological changes, restoring levels to near-control status. This indicates IDO activation promotes TLE progression, which is associated with modulation of the BDNF/TrkB signaling pathway, exacerbation of oxidative stress, and impairment of mitochondrial complex I/IV activities—supporting IDO as a potential therapeutic target for TLE.

## 1. Introduction

Temporal lobe epilepsy (TLE) is a common type of focal epilepsy characterized by recurrent seizures and progressive brain changes [1]. Abnormal discharges and neuronal damage often involve the medial temporal lobe structures, such as the hippocampus and amygdala [2], with the cornu ammonis 1 (CA1) region of the hippocampus being especially vulnerable to seizure-induced damage due to its high density of N-methyl-D-aspartate (NMDA) receptors and particular susceptibility to excitotoxic injury [3,4]. The resulting structural damage can have severe negative impacts on patients’ cognitive functions, emotions, and mental states, among other neuropsychological functions [5,6,7]. Currently, the treatment of TLE relies heavily on antiepileptic drugs, which mainly inhibit seizure activity, but about 30–40% of TLE patients are still resistant to existing drug treatments, manifesting drug-refractory epilepsy [8]. The occurrence and progression of TLE are influenced by a combination of factors, including neuroinflammation, metabolic imbalance, oxidative stress, neuronal damage, neurotransmitter abnormalities, blood–brain barrier dysfunction, neural network remodeling, genetic susceptibility and mitochondrial-related abnormalities [9,10,11]. These factors are intertwined and interact with each other to form a complex pathophysiological mechanism, making the pathogenesis and progression of TLE extremely complex.

Among these complex mechanisms, abnormalities in the tryptophan metabolic pathway are gradually gaining attention. The tryptophan metabolic pathway plays an important role in regulating the function of the nervous system and maintaining neurohomeostasis [12]. The kynurenine (KYN) pathway is the major metabolic pathway for tryptophan degradation [13]. Indoleamine 2,3-dioxygenase (IDO) is one of the rate-limiting enzymes in the tryptophan–kynurenine metabolic pathway [14], which is able to activate NMDA receptors and directly induce neuronal hyperexcitability through the generation of neurotoxic metabolites (e.g., quinolinic acid (QUIN)) [15]. Neurotoxic substances like QUIN can inhibit the development of vascular glucose transporters, induce reactive oxygen species (ROS)-mediated neurotoxicity, and disrupt mitochondrial function [12]. Targeting the KYN pathway attenuates mitochondrial dysfunction in a variety of diseases [16]. QUIN increases oxidative stress via nitric oxide synthase (nNOS) and NMDA, which leads to neurodegeneration [17]. Notably, aberrant activation of IDO is closely associated with dysregulation of the brain-derived neurotrophic factor (BDNF)/tyrosine kinase receptor B (TrkB) signaling pathway [18]. In epilepsy, BDNF exhibits dual roles, exerting both antiepileptic and pro-epileptic effects [19]. In physiological states, the BDNF/TrkB signaling pathway plays a critical role in neuroplasticity, neuronal survival, and cognitive function [20,21]; however, in chronic TLE, over-activation of this pathway may drive epileptic network formation by lowering seizure threshold and exacerbating glutamatergic excitotoxicity [22].

Based on the above research background, the present study aims to investigate the specific mechanisms by which IDO activation affects the BDNF/TrkB signaling pathway, oxidative stress, and mitochondrial respiratory chain complex activity. To this end, we used the IDO-specific inhibitor 1-Methyl-DL-Tryptophan (1-MT) to intervene in the pilocarpine-induced chronic TLE mouse model, and observed neuronal morphology and damage by combining Nissl staining and Fluoro-Jade B (FJB) fluorescent staining. Additionally, we used real-time quantitative PCR (qRT-PCR) and Western blot techniques to detect the mRNA and protein expression levels of IDO, BDNF, and TrkB. We also measured the levels of oxidative stress markers, including malondialdehyde (MDA), glutathione (GSH), and the activities of superoxide dismutase (SOD) and catalase (CAT), to comprehensively evaluate the oxidative stress status. Furthermore, we detected the activities of mitochondrial respiratory chain complexes I and IV to clarify the changes in mitochondrial respiratory chain complex activity. The study aims to reveal the multi-target mechanism of action of IDO activation in TLE and provide new theoretical basis for breaking the bottleneck of clinical treatment of TLE.

## 2. Materials and Methods

### 2.1. Experimental Animals

A total of 135 5- to 6-week-old Kunming (KM) male mice, weighing 30–35 g, were provided by Hunan SJA Laboratory Animal Co., Ltd. in Changsha, China (License No.: SCXK(Xiang)2019-0004). Kept in an air-conditioned animal house under specific pathogen-free (SPF) conditions, with an ambient temperature of 23 ± 1 °C, humidity of 50–70%, free feeding and drinking, and a 12 h light/12 h dark cycle. All procedures were performed in accordance with the Animal Research: Reporting of In Vivo Experiments (ARRIVE) guidelines [23] and were approved by the Animal Ethics Committee of Guangxi University of Chinese Medicine (Approval Code: DW20240603-150).

The 135 mice were acclimatized for 1 week and then randomly divided into two groups: the control group (*n* = 60) and the model group (*n* = 75). Mice in the model group were intraperitoneally injected with 2 mg/kg scopolamine methyl nitrate (TGI, Tokyo, Japan) and 2 mg/kg terbutaline hemisulfate salt (3ABIO, Shanghai, China), and 30 min later injected with 300 mg/kg pilocarpine hydrochloride (Sigma-Aldrich, St. Louis, MO, USA) to induce seizures. Pilocarpine was chosen as it stably induces TLE-like pathological features (e.g., hippocampal damage, spontaneous seizures) consistent with human TLE. According to the Racine grading criteria [24], grade IV or higher seizures were defined as status epilepticus (SE), and the seizures were recorded. For those that did not reach SE, an additional 150 mg/kg pilocarpine was administered every 30 min until SE occurred. After SE persisted for 3 h, the seizures were terminated by intraperitoneal injection of diazepam. In the control group, mice underwent identical procedures but received an equal volume of normal saline instead of pilocarpine hydrochloride to ensure consistency in handling and treatment. The model preparation was based on the literature [25].

**Figure 1:**
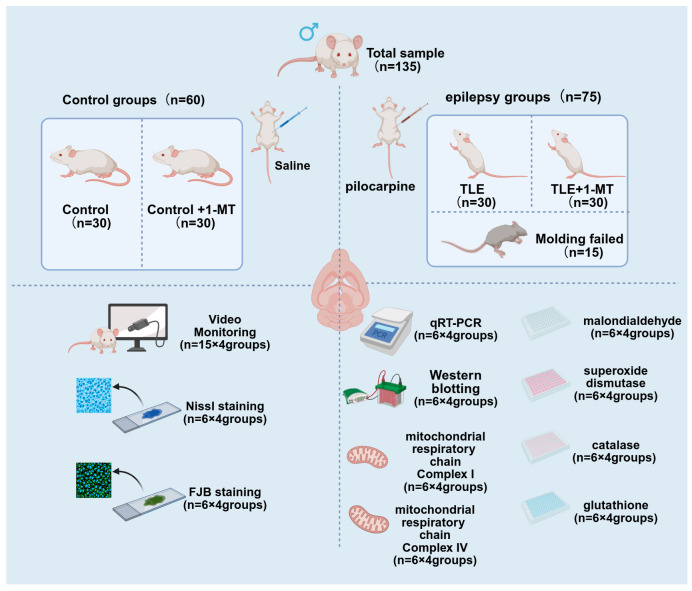
Diagram showing the distribution of animal samples. A total of 135 KM male mice were acclimatized for 1 week, then divided into control (*n* = 60) and model (*n* = 75) groups for TLE induction. After 15 model-group mice failed/ were excluded, 60 successful model mice formed TLE (*n* = 30) and TLE + 1-MT (*n* = 30) groups; the control group split into Control (*n* = 30) and Control + 1-MT (*n* = 30) groups. Drug administration started 14 days after SE, with 1-MT (50 mg/kg, twice daily) or saline for 2 weeks. The sample allocation for detections are as follows: 15 mice/group for seizure video monitoring; 6 mice/group for Nissl staining and FJB staining (neuronal damage); 6 mice/group for qRT-PCR and Western blot (IDO/BDNF/TrkB expression); 6 mice/group for oxidative stress indicators and mitochondrial complex activities assay.

During the model induction process, 15 mice in the model group died or were excluded due to failure to meet the seizure criteria. Consequently, 60 model mice successfully developed TLE and were randomly divided into two groups: the TLE group (*n* = 30) and the TLE + 1-MT group (*n* = 30). The control group was also divided into two groups: the Control group (*n* = 30) and the Control + 1-MT group (*n* = 30). Drug administration began 14 days after SE. This timing was chosen to allow the model to reach a stable phase of epilepsy, as previous studies have shown that seizure patterns and underlying pathological changes become more consistent during this period [26,27]. Mice in the TLE + 1-MT group and the control + 1-MT group were intraperitoneally injected with 50 mg/kg 1-MT solution [28] (Sigma-Aldrich, St. Louis, MO, USA) twice daily; the other groups were given an equal volume of normal saline, and the administration lasted for 2 weeks. (See Figure 1 for a detailed sample allocation plan).

### 2.2. Tissue Processing

After the completion of drug administration, the mice were anesthetized via intraperitoneal injection of 1% sodium pentobarbital (40 mg/kg). The depth of anesthesia was confirmed by the loss of righting reflex and pedal withdrawal reflex, after which mice were euthanized by cervical dislocation. The skull was immediately opened, and the whole brain was rapidly removed. For histological analysis, six mice from each group were selected, and their entire brains were fixed in 4% paraformaldehyde solution for subsequent Nissl and FJB staining. The remaining 24 mice from each group were used for molecular analysis. Their brains were rapidly dissected to isolate the hippocampal regions, which were then snap-frozen in liquid nitrogen and stored at −80 °C for subsequent Western blot and qRT-PCR analysis.

### 2.3. Video Monitoring

Following model establishment, eight mice from each group were randomly selected and promptly transferred to cages equipped with a video monitoring system. Continuous video recording was initiated immediately and maintained until euthanasia. Throughout the observation period, both the frequency of epileptic seizures and the duration of each seizure episode were meticulously documented for each group.

### 2.4. Nissl Staining

Following fixation in 4% formaldehyde, brain tissues were dehydrated through a graded ethanol series, cleared in xylene, and embedded in paraffin. Coronal sections (5 µm thick) were prepared and stained with 1% toluidine blue to evaluate neuronal morphology and injury, with a focus on the hippocampal CA1 region. To ensure transparency and reproducibility, six non-consecutive coronal sections were selected from the entire rostrocaudal extent of the hippocampal CA1 region for each mouse (*n* = 6 mice per group). These sections were examined under a light microscope. Qualitative assessment of neuronal morphology (soma integrity, Nissl body abundance) and injury was conducted by a single observer who was blinded to the experimental groups.

### 2.5. FJB Staining

Adjacent coronal sections (5 µm thick, prepared identically to Nissl-stained samples) were rinsed in phosphate-buffered saline (PBS), treated with 0.06% potassium permanganate for 15 min, and washed again in PBS. Sections were then incubated with FJB staining solution for 30 min at room temperature (protected from light), rinsed with distilled water, and air-dried. Finally, sections were cleared in xylene for 7 min and coverslipped with neutral gum. Six non-consecutive coronal sections, matching the rostrocaudal scope of the Nissl-stained sections, were analyzed per mouse (*n* = 6 mice per group). Staining results were visualized under the green channel of a fluorescence microscope and documented. The number of FJB-positive cells and total cells in the hippocampal CA1 region of each section was quantified using Aipathwell software (Version 2, Servicebio, Wuhan, China). A single observer, blinded to the experimental groups, verified the software-generated counts to ensure objectivity. The average positivity rate (FJB-positive cell count/total cell count) for the six sections per animal was calculated for subsequent group-wise statistical comparison.

### 2.6. qRT-PCR Analysis

Total RNA was extracted from the hippocampal tissues of mice using Trizol reagent (Invitrogen, Carlsbad, CA, USA). The concentration of RNA was measured using a Nanodrop 2000 spectrophotometer (Thermo Fisher Scientific, Waltham, MA, USA), and the purity of RNA was assessed by the A260/A280 absorbance ratio. Subsequently, RNA was reverse-transcribed into complementary DNA (cDNA) using the Primerscript Reverse Transcription kit (TaKaRa, Beijing, China). The qRT-PCR reactions were performed on a Light Cycler 480 system (Roche, Basel, Switzerland) using the TB Green Premix Ex Taq II kit (TaKaRa, Beijing, China). The total volume of the reaction mixture was 20 µL, and the reaction conditions were as follows: pre-denaturation at 95 °C for 30 s; followed by 40 cycles of amplification (95 °C for 5 s, 60 °C for 30 s); and finally, a melting curve analysis (95 °C for 5 s, 60 °C for 60 s, 95 °C for 15 s). β-Actin was used as the internal reference gene (Sangon Biotech, Shanghai, China), and the relative expression levels of the target genes were calculated using the 2^−ΔΔCt^ method [29]. The primers were synthesized by Gene Create (Gene Create Biotech, Wuhan, China), and the sequences are detailed in Table 1.

### 2.7. Western Blot Assay

Hippocampal tissues from mice were homogenized using radio-immunoprecipitation assay (RIPA) lysis buffer (Beyotime, Shanghai, China) with the addition of protease and phosphatase inhibitors to prevent protein degradation. Protein concentration was determined using a Bicinconinic Acid Protein Assay Kit (Beyotime, Shanghai, China). A total of 30 μg of protein from each sample was subjected to sodium dodecyl sulfate-polyacrylamide gel electrophoresis (SDS-PAGE) using a Bio-Rad Mini-PROTEAN Tetra vertical electrophoresis cell equipped with a PowerPac Basic power supply (Bio-Rad, Hercules, CA, USA). Resolved proteins were then transferred onto Immobilon-P polyvinylidene fluoride (PVDF) membranes (Merck Millipore, Darmstadt, Germany) via a Bio-Rad Mini Trans-Blot electrophoretic transfer cell (Bio-Rad, Hercules, CA, USA) under constant voltage conditions.

The membranes were blocked with 5% skim milk at room temperature for 2 h and then incubated with rabbit anti-mouse IDO primary antibody (1:1000, Proteintech, Wuhan, China) at 4 °C overnight. After washing three times with Tris-buffered saline with Tween 20 (TBST), the membranes were incubated with horseradish peroxidase (HRP)-conjugated goat anti-mouse secondary antibody (1:5000, Beyotime, Shanghai, China) at room temperature for 1 h. The proteins were visualized using an enhanced chemiluminescence (ECL) detection kit (Biosharp, Anhui, China). Chemiluminescent signals were captured using a Tanon 5200 Multi Imaging System (Tanon, Shanghai, China). The band densities were analyzed using ImageJ 1.54g software (National Institutes of Health, Bethesda, MD, USA), with β-actin serving as an internal reference to calculate the relative expression levels of IDO protein.

### 2.8. Determination of SOD, MDA, CAT, and GSH Levels

To determine the levels of MDA, SOD, Reduced GSH and CAT in the hippocampal tissue of mice, we used detection kits provided by Solarbio, Beijing, China, with catalog numbers BC0025, BC0175, BC1175, and BC0205, respectively. All experimental procedures were strictly carried out according to the instructions provided with each kit. After sample processing and preparation of the reaction system, the absorbance values were measured using a full-wavelength microplate reader (Thermo Fisher Scientific, Waltham, MA, USA) at the characteristic wavelengths corresponding to each indicator.

### 2.9. Determination of Mitochondrial Respiratory Chain Complex I and IV Activities

To determine the activity of mitochondrial respiratory chain Complex I and Complex IV in the hippocampal tissue of mice, we used detection kits provided by Solarbio, Beijing, China, with catalog numbers BC0515 and BC0945, respectively. After the mice completed the drug intervention and were euthanized under anesthesia, their brains were removed, and the hippocampal tissues were dissected on an ice-cold surface. The tissues were homogenized and then centrifuged at 600× *g* for 10 min at 4 °C, followed by centrifugation at 11,000× *g* for 15 min to separate the precipitate containing the target complexes. Subsequently, according to the kit instructions, reagents were added to the precipitate and treated with ultrasonication to release enzyme activity. Finally, the activity of complexes I and IV was assessed by measuring the change in absorbance by a full-wavelength microplate reader.

### 2.10. Statistical Analysis

All statistical analyses were performed using GraphPad Prism 10.3.0 (San Diego, CA, USA). The normality of all datasets was confirmed using the Shapiro–Wilk test. Data are presented as mean ± SEM. Comparisons between two groups were made using an unpaired two-tailed *t*-test (for data with homogeneous variances). Multiple comparisons were analyzed by one-way Analysis of Variance (ANOVA) followed by Tukey’s post hoc test (for data with equal variances) or Welch’s ANOVA followed by the Games–Howell test (for data with heterogeneous variances). *p* < 0.05 was considered statistically significant.

## 3. Results

### 3.1. Effect of IDO Activation on Seizure Behavior in Mice

Video monitoring analysis indicated that IDO activation significantly exacerbated seizure behavior in TLE model mice. Compared with the control group, in which no seizures were detected, mice in the TLE group exhibited typical spontaneous recurrent seizures. Further investigation revealed that intervention with the IDO-specific inhibitor 1-MT significantly reduced seizure frequency (*p* < 0.05) and shortened seizure duration (*p* < 0.05). These results suggest that IDO activation significantly promotes seizures in the TLE mouse model, and that inhibiting IDO activity can effectively mitigate the severity of seizures, indicating that IDO plays a crucial role in the pathogenesis of TLE (Table 2).

### 3.2. IDO Activation Leads to Neuronal Damage

**Figure 2:**
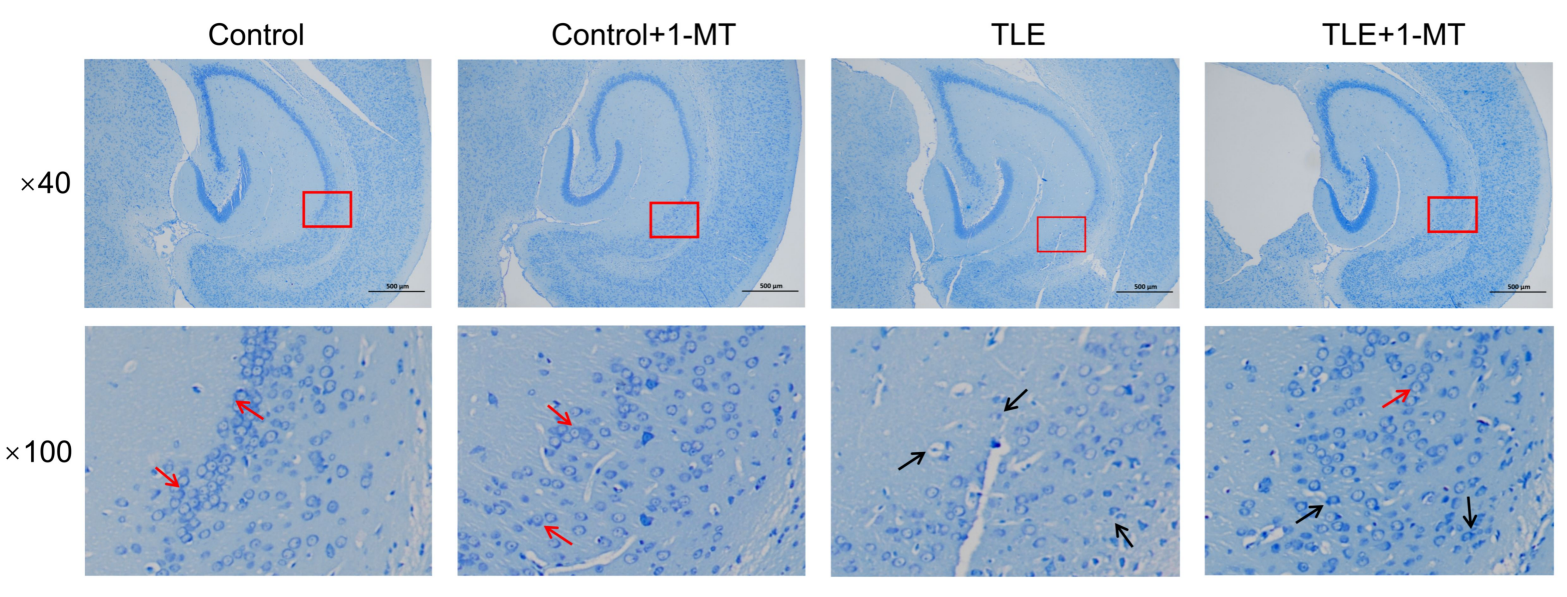
IDO inhibition alleviates TLE-induced neuronal morphological damage in the hippocampal CA1 region. Nissl staining was used to examine neuronal integrity in the hippocampal CA1 region. Compared with controls, TLE mice showed fewer Nissl bodies, while 1-MT treatment increased their abundance, suggesting a neuroprotective effect. In representative images, red arrows indicate neurons with abundant Nissl bodies (normal morphology), and black arrows indicate neurons with reduced or abnormal Nissl bodies (TLE-induced damage). Areas within red boxes (imaged at ×40 magnification) are shown at higher magnification (×100) to illustrate cellular details; scale bar = 500 μm; *n* = 6. TLE: temporal lobe epilepsy; 1-MT: 1-Methyl-DL-tryptophan.

Nissl staining analysis demonstrated that within the hippocampal CA1 region, neurons in the control and control + 1-MT groups displayed uniform soma size, intact morphological features, and organized arrangement. In striking contrast, the TLE group exhibited a significant reduction in hippocampal neuron count, disorganized cellular alignment, diminished Nissl staining intensity in neuronal soma, reduced Nissl body abundance, and prominent nuclear pyknosis or fragmentation. Relative to the control + 1-MT group, neuronal damage was alleviated in the TLE + 1-MT group, characterized by partial preservation of neuronal morphology in some cells and an increase in Nissl body quantity (Figure 2).

**Figure 3:**
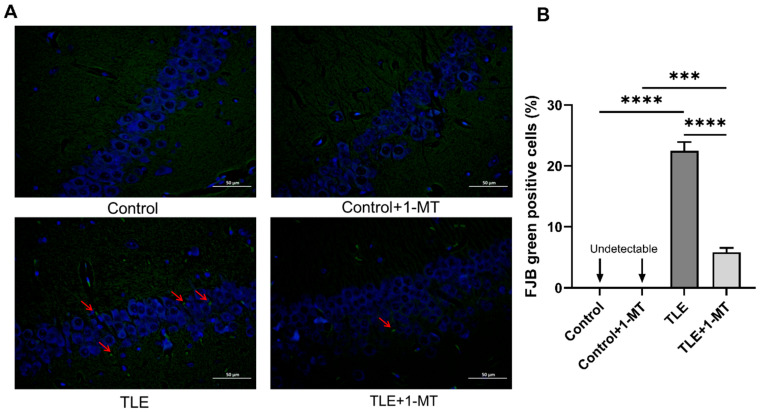
IDO inhibition reduces TLE-induced neuronal degeneration in the hippocampal CA1 region. (**A**) Fluoro-Jade B (FJB) staining of hippocampal CA1 region showing green fluorescence in FJB-positive cells. The TLE group has a marked increase in green fluorescence compared to controls, with red arrows pointing to FJB-positive cells (degenerating neurons). The TLE + 1-MT group shows reduced fluorescence, indicating less neuronal damage (Scale bar: 50 µm, *n* = 6). (**B**) Bar graph representing the percentage of FJB-positive cells among total cells. The TLE group has a significantly higher FJB positivity rate than the control group. After intervention with 1-MT, the TLE + 1-MT group shows a reduced FJB positivity rate. TLE: temporal lobe epilepsy; 1-MT: 1-Methyl-DL-Tryptophan. Data are presented as mean ± SEM (*n* = 6). Statistical testing was performed using one-way ANOTuky’s post hoc test. *** *p* < 0.001, **** *p* < 0.0001. TLE: temporal lobe epilepsy; 1-MT: 1-Methyl-DL-Tryptophan.

These morphological observations were further validated using FJB staining—a specific marker for degenerating neurons. For FJB staining, the observation was also focused on the hippocampal CA1 region. No overt FJB-positive cells (green, fluorescent signals) were detected in the control or control + 1-MT groups, whereas numerous distinct FJB-positive cells with intense green fluorescence were readily detectable in both the TLE and TLE + 1-MT groups (Figure 3A). Quantitative analysis of FJB-positive cells revealed a significantly higher positivity rate of FJB-positive cells in the TLE group compared to the control group (*p* < 0.05), indicative of severe neuronal injury. Notably, following intervention with the IDO-specific inhibitor 1-MT, the TLE + 1-MT group showed a significant reduction in positivity rate of FJB-positive cells relative to the TLE group (*p* < 0.05), confirming that inhibition of IDO activation effectively mitigates neuronal damage and degeneration (Figure 3). However, the positivity rate of FJB-positive cells in the TLE + 1-MT group remained elevated compared to that in the control + 1-MT group (Figure 3B).

### 3.3. Expression Changes in IDO in the Hippocampus of TLE Model Mice

**Figure 4:**
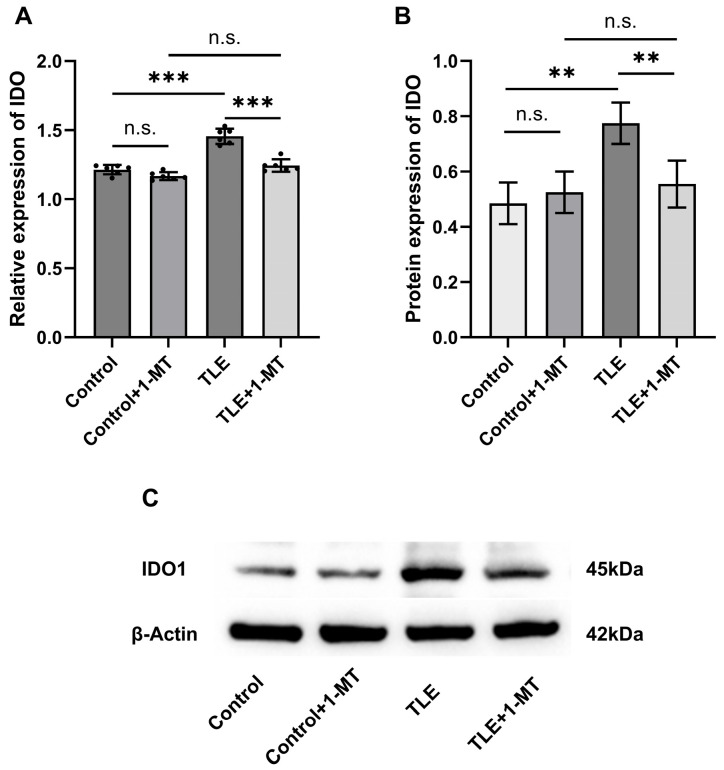
IDO expression is upregulated in the hippocampus of TLE model mice and normalized by 1-MT intervention. Seizures can activate IDO expression. (**A**) The relative expression level of ItingDO mRNA in the hippocampus of TLE mice increased. (**B**) The expression level of IDO protein in the hippocampus of TLE mice increased. (**C**) Representative Western blot results of IDO in the mouse hippocampus. Data are presented as mean ± SEM (*n* = 6). Statistical testing was performed using one-way ANOVA. ** *p* < 0.01, *** *p* < 0.001, n.s. = not significant. TLE: temporal lobe epilepsy; IDO: Indoleamine 2,3-Dioxygenase; 1-MT: 1-Methyl-DL-Tryptophan.

In the hippocampal tissue of TLE model mice, IDO expression was sigificantly altered, and closely related to the pathological process of epilepsy. qRT-PCR analysis showed no significant difference between the control and control + 1-MT groups (*p* > 0.05). However, compared with the control group, the mRNA level of IDO in the hippocampus of TLE mice was significantly upregulated (*p* < 0.05), indicating IDO activation in epilepsy. After 1-MT intervention, the mRNA level of IDO in the hippocampus of TLE + 1-MT mice was significantly downregulated compared with the TLE group (*p* < 0.05), with no significant difference between the TLE + 1-MT and control + 1-MT groups (*p* > 0.05) (Figure 4A). Western blot results were consistent with the mRNA changes. Compared with the control group, the protein level of IDO in the hippocampus of TLE mice was significantly increased (*p* < 0.05), while the protein level of IDO in TLE + 1-MT mice was significantly decreased compared with the TLE group (*p* < 0.05), with no statistical difference between the TLE + 1-MT and control + 1-MT groups (*p* > 0.05) (Figure 4B). These results indicate that IDO expression is significantly upregulated in the TLE model, and 1-MT intervention can effectively inhibit IDO activation, restoring it to the control level.

### 3.4. Overexpression of the BDNF/TrkB Pathway in the Hippocampus of TLE Model Mice Is Associated with IDO Activation

**Figure 5:**
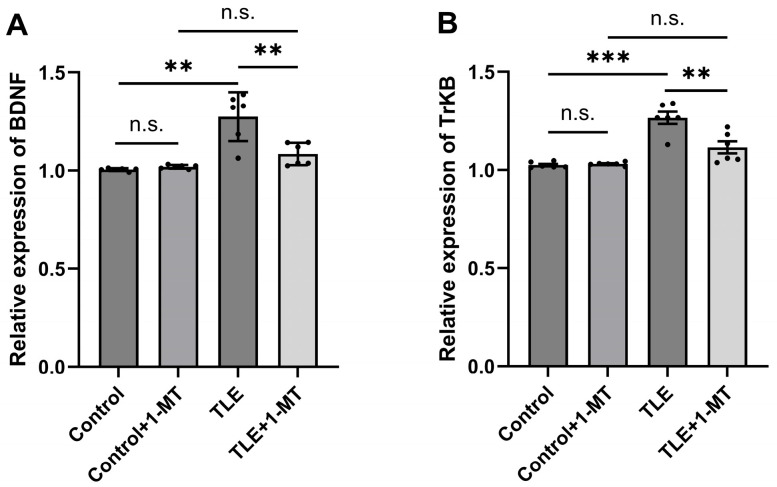
IDO inhibition is associated with reduced overexpression of BDNF/TrkB pathway in the hippocampus of TLE model mice. The expression levels of BDNF and TrkB genes in the hippocampal tissue of TLE mice were significantly elevated. 1-MT intervention demonstrated an inhibitory effect on the overexpression of the BDNF/TrkB signaling pathway. (**A**) Increased expression of BDNF mRNA and (**B**) TrkB mRNA in the hippocampus of TLE mice. Data are presented as mean ± SEM (*n* = 6). Statistical analysis was performed using one-way ANOVA. ** *p* < 0.01, *** *p* < 0.001, n.s. = not significant. BDNF: brain-derived neurotrophic factor; TrkB: Tropomyosin-Related Kinase B; TLE: temporal lobe epilepsy; 1-MT: 1-Methyl-DL-Tryptophan.

qRT-PCR results showed that compared with the control group, the mRNA expression levels of BDNF and its receptor TrkB in the hippocampal tissue of TLE model mice were significantly increased (*p* < 0.05), indicating that seizures may upregulate the activity of the BDNF/TrkB signaling pathway. However, after intervention with 1-MT, the mRNA expression levels of BDNF (Figure 5A) and TrkB (Figure 5B) in the hippocampus of TLE + 1-MT mice were significantly decreased compared with the TLE model group (*p* < 0.05), and showed no significant difference compared to the Control + 1-MT group (*p* > 0.05), suggesting that inhibiting IDO activation may exert antiepileptic effects, which is accompanied by suppression of the BDNF/TrkB signaling pathway. These results further support the important role of IDO activation in regulating molecular mechanisms related to neuroplasticity.

### 3.5. IDO Activation Induces Oxidative Stress Injury in TLE Model Mice

**Figure 6:**
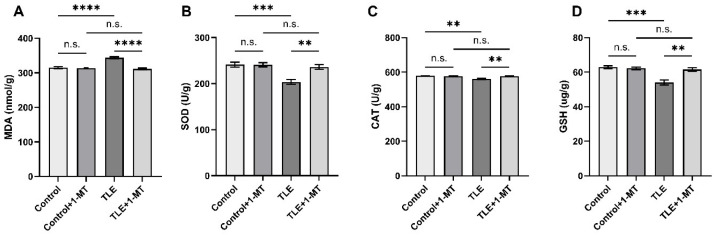
IDO inhibition mitigates TLE-induced oxidative stress injury, as evidenced by changes in MDA levels and antioxidant indices. The results were normalized to total protein content. Compared with the control group, the TLE group showed increased oxidative stress marker (MDA) and decreased antioxidant markers (SOD, CAT, GSH). (**A**) MDA levels in the hippocampus; (**B**) SOD activity in the hippocampus; (**C**) CAT activity in the hippocampus; (**D**) GSH content in the hippocampus. Data are presented as mean ± SEM (*n* = 6). Significance was determined using one-way ANOVA, ** *p* < 0.01, *** *p* < 0.001, **** *p* < 0.0001, n.s. = not significant. MDA: Malondialdehyde; SOD: Superoxide Dismutase; CAT: Catalase; GSH: Glutathione; TLE: temporal lobe epilepsy; 1-MT: 1-Methyl-DL-Tryptophan.

In TLE model mice, the levels of oxidative stress were significantly elevated, as evidenced by significantly higher MDA levels compared to the control group (*p* < 0.05) (Figure 6A), while the activities of the antioxidant function indicators SOD (Figure 6B) and CAT (Figure 6C), as well as the GSH content (Figure 6D), were all significantly lower than those in the control group (*p* < 0.05), indicating that TLE can lead to a decrease in antioxidant capacity. In contrast, after intervention with 1-MT, the MDA content in the TLE + 1-MT group was significantly reduced, while the activities of SOD and CAT, as well as the content of GSH, were all significantly higher than those in the TLE group. Furthermore, there were no significant differences in the aforementioned indicators between the TLE + 1-MT group and the control + 1-MT group after 1-MT intervention (*p* > 0.05). These results demonstrate that IDO activation leads to oxidative stress injury, and that inhibiting IDO activation can significantly ameliorate the oxidative stress injury induced by TLE.

### 3.6. Impairment of Mitochondrial Complex Activities Induced by IDO Activation

**Figure 7:**
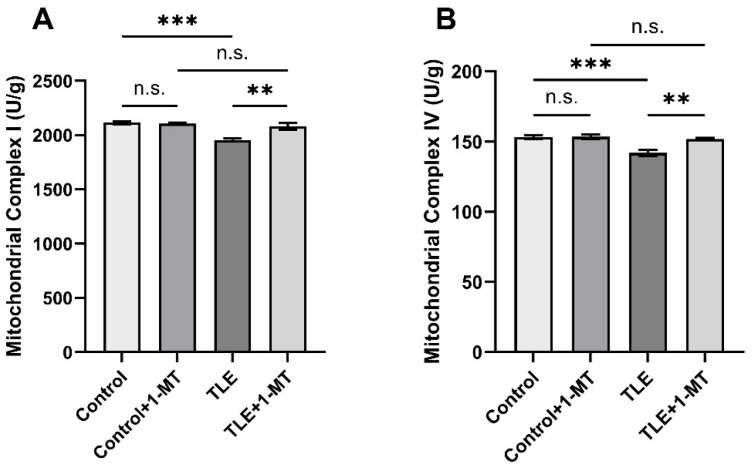
IDO inhibition ameliorates TLE-induced deficits in mitochondrial Complex I and IV activities. The results were normalized to total protein content. Compared with the control group, the activities of mitochondrial Complex I and IV in TLE mice were significantly decreased. The mitochondrial activity was increased in the TLE + 1-MT group. (**A**) Changes in mitochondrial Complex I activity; (**B**) Changes in mitochondrial Complex IV activity. Data are presented as mean ± SEM (*n* = 6). Significance was determined by one-way ANOVA, ** *p* < 0.01, *** *p* < 0.001, n.s. = not significant. TLE: temporal lobe epilepsy; 1-MT: 1-Methyl-DL-Tryptophan.

During the assessment of mitochondrial respiratory chain complex activity, the activities of mitochondrial Complex I (Figure 7A) and IV (Figure 7B) in the TLE group were significantly lower than those in the control group (*p* < 0.05), highlighting the impairment of mitochondrial respiratory chain complex activity by TLE, which may affect neuronal energy metabolism and overall health. However, the activities of Complex I and IV in the TLE + 1-MT group were significantly higher than those in the TLE group (*p* < 0.05), and showed no significant difference compared to the control + 1-MT group (*p* > 0.05), indicating that inhibiting IDO activation can effectively ameliorate the TLE-induced deficit in mitochondrial respiratory chain complex activities.

## 4. Discussion

### 4.1. Main Findings

This study systematically reveals the multi-target mechanism of action of IDO activation in the pathogenesis of TLE. Our findings demonstrate that aberrant activation of IDO significantly exacerbates seizure frequency and duration, promotes neuronal damage, is associated with upregulation of the BDNF/TrkB signaling pathway, induction of oxidative stress, and impairment of mitochondrial respiratory chain complex activity in a pilocarpine-induced TLE mouse model. Crucially, inhibition of IDO with 1-MT effectively reversed these pathological changes, restoring neuronal integrity, reducing seizure burden, normalizing BDNF/TrkB expression, alleviating oxidative stress, and ameliorating deficits in mitochondrial complex activities. These results collectively indicate that IDO activation plays a central role in promoting epileptogenesis through multi-faceted mechanisms, highlighting its potential as a therapeutic target for TLE.

### 4.2. Comparisons with Previous Studies

Previous studies have confirmed IDO activation through KYN/Tryptophan (TRP) and 5-Hydroxytryptamine (5-HT)/TRP ratio measurements [30,31], thus the current investigation focused on evaluating IDO activation at transcriptional and translational levels using qRT-PCR and Western blot analyses rather than repeating metabolic measurements.

Existing evidence has emphasized the critical role of IDO in neuroinflammation and its relevance to various neurological disorders [32,33]. Our previous work has specifically elucidated this mechanism in TLE, demonstrating that the upregulation of hippocampal pro-inflammatory cytokines Interleukin-1 beta (IL-1β) and Interleukin-6 (IL-6) activates IDO expression, which in turn alters tryptophan metabolism [28]. In TLE, IDO acts as a key initiator that links inflammation and excitotoxicity pathways [34], drving disease progression mainly through the following mechanisms: IDO catalyzes the metabolism of tryptophan to generate neurotoxic metabolites (such as 3-hydroxykynurenine and QUIN). These metabolites, by activating the NMDA receptor, induce neuronal hyperexcitability, which in turn exacerbates epileptic seizures and neuronal damage [35]. Additionally, the initial activation of IDO triggers a series of downstream events, including the induction of oxidative stress and the reduction in inhibitory neurotransmitter synthesis (such as gamma-aminobutyric acid (GABA), which further propagate the disease process [36,37].

### 4.3. Possible Mechanisms

This multifaceted role of IDO in initiating and driving the disease process is supported by several studies. For instance, research has found that inhibiting IDO1 can reduce the production of neurotoxic metabolites, thereby suppressing seizures and alleviating neuronal damage [30]. In neuroinflammatory models, the activation of IDO is positively correlated with the levels of pro-inflammatory cytokines, and inhibiting IDO activity can mitigate inflammation-mediated neuronal injury [38]. These findings underscore the importance of IDO as an upstream regulator in the pathogenesis of TLE, highlighting its potential as a therapeutic target.

Notably, kynurenine pathway metabolites such as 3-hydroxykynurenine (3-HK) and QUIN are known to induce oxidative stress through multiple mechanisms, including direct generation of ROS and depletion of antioxidant defenses [39,40]. Specifically, QUIN can activate NMDA receptors leading to calcium influx and subsequent mitochondrial dysfunction, further exacerbating ROS production [41,42]. Our findings of elevated MDA and reduced SOD, CAT, and GSH levels in TLE mice are consistent with these reports, suggesting a link between IDO-driven kynurenine pathway activation and oxidative damage.

As a key regulatory protein for central nervous system (CNS) function, BDNF plays an important role in neuroplasticity, neuronal survival and synaptic remodeling [43]. Under physiological conditions, BDNF promotes neuronal growth, differentiation, and synaptic stability by activating its receptor TrkB, which is crucial for maintaining neurohomeostasis [44]. However, in TLE, the abnormal upregulation of BDNF expression may lead to neuronal overexcitation, lower the seizure threshold, and exacerbate the progression of epilepsy [45].This “double-edged sword” effect makes the role of the BDNF/TrkB signaling pathway in epilepsy complex. This paradoxical role of BDNF presents a significant therapeutic challenge, as complete inhibition of its signaling could impair beneficial neuroplasticity while unabated activity may promote hyperexcitability. Our findings suggest that targeting upstream IDO activation with 1-MT offers a strategic approach to this dilemma. By mitigating the initial pathological trigger (e.g., neurotoxic kynurenine metabolites and oxidative stress), 1-MT intervention indirectly normalized the aberrant BDNF/TrkB overexpression observed in TLE mice, ultimately resulting in reduced seizures and neuronal damage. This indicates that modulating IDO activation, an upstream event, may help restore balance in the BDNF/TrkB pathway, rather than direct inhibition, and may represent a more nuanced and potentially safer therapeutic strategy for TLE. It proposes a novel concept of treating neural excitability not by suppressing neural plasticity altogether, but by correcting the underlying pathological drive that disrupts its equilibrium.

This may be because in TLE, IDO metabolites such as QUIN, a potent NMDA receptor agonist, can activate NMDA receptors, leading to elevated intracellular calcium levels. This calcium influx may activate multiple calcium-dependent signaling pathways, including the Calcium/Calmodulin-Dependent Protein Kinase II (CaMKII/CREB) pathway, thereby modulating BDNF gene transcription [46,47]. Furthermore, excessive NMDA receptor activation may trigger pro-inflammatory pathways such as Nuclear Factor-Kappa B (NF-κB) signaling, which could further influence BDNF/TrkB signaling [48,49]. Alternatively, tryptophan pathway metabolites may indirectly affect BDNF/TrkB through microglial activation and subsequent release of cytokines (e.g., IL-1β, Tumor Necrosis Factor-alpha (TNF-α)), which have been demonstrated to regulate BDNF expression under neuroinflammatory conditions [50].

Studies have shown that oxidative stress persists in the development of epilepsy and is positively correlated with disease severity [51,52]. Oxidative stress further affects normal celludar function by increasing the production of free radicals and damaging cell membranes, proteins, and DNA [53]. Impaired mitochondrial complex activity, which serves as the cell’s energy factory, leads to abnormal cellular energy metabolism and exacerbates neuronal damage [54]. Oxidative stress and mitochondrial-related functional abnormalities are recognized as key factors in neuronal injury in epilepsy. On the one hand, mitochondria represent a major source of ROS [55].Impairment of the mitochondrial electron transport chain leads to increased electron leakage and enhanced generation of superoxide anions, thereby initiating or exacerbating oxidative stress [56,57].On the other hand, excessive ROS further damages mitochondrial components—including membrane lipids, proteins, and mitochondrial DNA (mtDNA)—compromising their structural integrity and function [51]. This results in reduced adenosine triphosphate (ATP) synthesis, disrupted calcium homeostasis, and activation of apoptotic pathways [58]. Our study revealed that IDO activation not only induced oxidative stress markers (elevated MDA and decreased SOD, CAT, and GSH levels) but also caused impairments in the activities of mitochondrial complexes I and IV. Thus, IDO activation may drive epileptogenesis, which involves the production of neurotoxic metabolites such as QUIN and is accompanied by oxidative stress and mitochondrial dysfunction, which collectively contribute to the progression of epilepsy. Our study shows that inhibition of IDO activation reduces oxidative stress by decreasing MDA levels and increasing SOD, CAT and GSH levels. This is consistent with previous studies that emphasized the importance of reducing oxidative stress in attenuating neuronal injury in epilepsy [59,60,61]. In addition, we found that inhibition of IDO activation ameliorated the activity deficits of mitochondrial respiratory chain complexes I and IV, a finding that echoes previous studies: mitochondrial abnormalities is directly involved in excitotoxicity-induced neuronal death and is strongly associated with epilepsy-related cognitive deficits [62,63] whereas targeted antioxidant interventions (e.g., hydrogen therapy) can indirectly inhibit epileptic networks by attenuating hippocampal oxidative damage [64].

### 4.4. Clinical Implications

Our study provides robust preclinical evidence positioning IDO as a novel and promising therapeutic target for TLE. The multifaceted benefits of 1-MT intervention—reducing seizure burden, mitigating neuronal damage, quenching oxidative stress, restoring mitochondrial function, and normalizing aberrant BDNF/TrkB signaling—suggest that IDO inhibition offers a comprehensive therapeutic strategy that extends beyond mere symptom control. This is particularly relevant for the 30–40% of TLE patients who are refractory to current antiseizure medications, which primarily target ion channels or neurotransmitters. Our data imply that IDO inhibitors could function as a disease-modifying therapy, potentially altering the progressive course of TLE by disrupting the vicious cycle of neuroinflammation, excitotoxicity, and metabolic dysfunction. Furthermore, the association between IDO activation and neuropsychiatric comorbidities in epilepsy suggests that such a strategy might also confer benefits for cognitive and emotional deficits, offering a more holistic treatment approach. Future clinical translation could involve patient stratification based on neuroinflammatory or metabolic biomarkers to identify those most likely to benefit from IDO-targeted therapies.

### 4.5. Limitations

The present study systematically investigated the effects of IDO activation on TLE across multiple dimensions, including seizure behavior, neuronal injury, BDNF/TrkB signaling, oxidative stress, and mitochondrial respiratory chain complex activity. This comprehensive assessment of IDO’s multifaceted roles addresses previous gaps in single-mechanism studies. Several limitations should be acknowledged: First, while we observed cognitive and mood impairments characteristic of TLE [65], the direct impact of IDO activation on these neuropsychiatric dimensions remains undetermined. Second, this study did not directly measure IDO enzymatic activity or kynurenine pathway metabolites, which would have provided more direct evidence of pathway activation. Third, the current study did not include direct measurements of neuroinflammatory markers, such as microglial activation or cytokine profiles, which are upstream regulators of IDO and important components of TLE pathophysiology. Fourth, and critically, our study employed systemic (intraperitoneal) administration of 1-MT. While effective, this approach inhibits IDO throughout the body. Given that IDO is a pivotal regulator of immune tolerance in peripheral tissues, its systemic inhibition carries a potential risk of disrupting immune homeostasis, potentially triggering or exacerbating autoimmune conditions or immune-related adverse events, as observed in oncology trials with IDO inhibitors. Our study did not evaluate these potential immunological sequelae. Fifth, the assessment of mitochondrial integrity was limited to the enzymatic activities of complexes I and IV, and did not include more direct functional readouts such as mitochondrial membrane potential, ATP production, or ROS levels. Sixth, although β-actin expression was stable across groups in our experimental setting, the use of a single reference gene remains a limitation. Finally, due to the complex dual properties of BDNF—specifically its ability to both promote seizures and exert neuroprotective effects—no definitive conclusions have yet been reached regarding the specific functional outcomes caused by IDO-mediated upregulation of the BDNF/TrkB signaling pathway in our established model.

### 4.6. Future Research Directions

Based on the current findings, we propose the following key research directions: (1) In-depth study of the mechanism of action of IDO activation in TLE, especially its relationship with cognitive and emotional functions; (2) Direct measurement of IDO enzymatic activity and kynurenine pathway metabolites to provide more conclusive evidence of IDO pathway activation in TLE; (3) Combining the detection of neuroinflammatory markers (e.g., microglia activation markers, cytokine levels, etc.) to further explore the modulatory effects of IDO activation on TLE-associated neuroinflammation; (4) To mitigate the potential systemic immunological risks, future work should prioritize the development of brain-targeted IDO delivery systems, such as nanoparticle encapsulation or prodrugs, to maximize central efficacy while minimizing peripheral exposure and off-target immune effects; (5) Comprehensive evaluation of mitochondrial function—including membrane potential, ATP production, ROS levels, and ultrastructural analysis—to better understand the impact of IDO activation on mitochondrial health; (6) Utilization of multiple validated reference genes in qRT-PCR analyses to improve the robustness of gene expression data; (7) Conducting an interaction between IDO and BDNF/TrkB signaling pathway in-depth studies to elucidate its role in the pathogenesis of TLE and to identify more effective therapeutic targets; (8) Long-term safety studies are essential to evaluate the consequences of chronic IDO inhibition on immune system function and the incidence of autoimmunity in pre-clinical models; and (9) Considering the significant role of oxidative stress in the development of TLE and the fact that mitochondria are a main source of ROS in the cell, we will also investigate the potential of combining 1-MT with mitochondria-targeted antioxidants to enhance the therapeutic efficacy against oxidative stress in TLE.

## 5. Conclusions

This study suggests that IDO activation is a key upstream driver in the development of TLE, significantly influencing its progression through multiple mechanisms. Specifically, IDO activation may contribute to epilepsy progression, potentially through mechanisms involving the BDNF/TrkB signaling pathway, oxidative stress, and mitochondrial respiratory chain activity. These findings provide an important basis for the development of novel multi-targeted antiepileptic strategies, but the mechanistic complexity and safety challenges still need to be overcome before they can be applied clinically. Future studies should aim to reveal the precise action network of IDO and promote its translational application in epilepsy.

## Figures and Tables

**Table 1:** Primer sequence.

Gene	Primer Sequence (5′ to 3′)
IDO	Forward: GTGTGTGAATGGTCTGGTCTCTGTG Reverse: ACATTTGAGGGCTCTTCCGACTTG
BDNF	Forward: TGACAGTATTAGCGAGTGGG Reverse: GCAGCCTTCCTTGGTGTGTGTA
TrkB	Forward: CGCTTCAGTGGTTCTACA Reverse: CCTTCCCATACTCGTTCTT
β-Actin	Forward: GTGCTATGTTGCTCTAGACTTCG Reverse: ATGCCACAGGATTCCATACC

All primer sequences were designed using NCBI Primer-BLAST tool(Primer3 and BLAST+ 2.16.0; National Center for Biotechnology Information, Bethesda, MD, USA) and validated for specificity.

**Table 2:** Daily Seizure Frequency and Duration in Each Group of Mice (*n* = 8).

Group	Seizure Frequency (Times/Day)	Duration (s)
Control Group	ND	ND
Control + 1-MT Group	ND	ND
TLE Group	5 ± 0.38	58.68 ± 2.78
TLE + 1-MT Group	3.63 ± 0.38 *	39.13 ± 1.88 ****

Data are normally distributed and expressed as Mean ± SEM. Comparisons between groups were made using an unpaired *t*-test. * *p* < 0.05, **** *p* < 0.0001 vs. TLE group, ND: No seizures detected, 1-MT: 1- methyltryptophan, TLE: temporal lobe epilepsy.

## Data Availability

The original contributions presented in this study are included in the article. Further inquiries can be directed to the corresponding author.

## Abbreviations

The following abbreviations are used in this manuscript:

IDO	Indoleamine 2,3-Dioxygenase
BDNF	Brain-Derived Neurotrophic Factor
TrkB	Tyrosine Kinase B
TLE	Temporal Lobe Epilepsy
1-MT	1-Methyl-DL-Tryptophan
ARRIVE SE	Animal Research: Reporting of In Vivo Experiments Status Epilepticus
qRT-PCR	Quantitative Real-Time Polymerase Chain Reaction
cDNA RIPA NMDA	complementary DNA Radio-Immunoprecipitation Assay N-methyl-D-aspartate
FJB	Fluoro-Jade B
MDA	Malondialdehyde
GSH	Glutathione
SOD	Superoxide Dismutase
CAT	Catalase
SDS-PAGE	Sodium Dodecyl Sulfate-Polyacrylamide Gel Electrophoresis
PVDF	Polyvinylidene Fluoride
TBST	Tris-Buffered Saline with Tween 20
HRP	Horseradish Peroxidase
ECL	Enhanced Chemiluminescence
SPF	Specific Pathogen-Free
ANOVA	Analysis of Variance
NMDA CA1	N-Methyl-D-Aspartate Cornu Ammonis 1
KYN TRP 5-HT	Kynurenine Tryptophan 5-Hydroxytryptamine
QUIN	Quinolinic Acid
GABA 3-HK	Gamma-Aminobutyric Acid 3-hydroxykynurenine
CNS IL-6	Central Nervous System Interleukin-6
IL-1β	Interleukin-1β
TNF-α	Tumor Necrosis Factor-α
CaMKII	Calcium/Calmodulin-Dependent Protein Kinase II
CREB mtDNA	cAMP Response Element-Binding Protein mitochondrial DNA
NF-κB	Nuclear Factor-Kappa B
KM	Kunming (mouse strain)
ATP	Adenosine Triphosphate
ROS nROS	Reactive Oxygen Species neuronal Nitric Oxide Synthase
PBS	Phosphate-Buffered Saline
